# Greenhouse gas performance of biochemical biodiesel production from straw: soil organic carbon changes and time-dependent climate impact

**DOI:** 10.1186/s13068-017-0907-9

**Published:** 2017-09-13

**Authors:** Hanna Karlsson, Serina Ahlgren, Mats Sandgren, Volkmar Passoth, Ola Wallberg, Per-Anders Hansson

**Affiliations:** 1 Department of Energy and Technology, Swedish University of Agricultural Sciences, Uppsala, Sweden; 2 Department of Chemical Engineering, Lund University, Lund, Sweden; 3 Department of Molecular Sciences, Swedish University of Agricultural Sciences, Uppsala, Sweden

**Keywords:** Life cycle assessment, FAME, Biorefinery, Oleaginous yeast, Lignocellulosic biomass

## Abstract

**Background:**

Use of bio-based diesel is increasing in Europe. It is currently produced from oilseed crops, but can also be generated from lignocellulosic biomass such as straw. However, removing straw affects soil organic carbon (SOC), with potential consequences for the climate impact of the biofuel. This study assessed the climate impacts and energy balance of biodiesel production from straw using oleaginous yeast, with subsequent biogas production from the residues, with particular emphasis on SOC changes over time. It also explored the impact of four different scenarios for returning the lignin fraction of the biomass to soil to mitigate SOC changes. Climate impact was assessed using two methods, global warming potential (GWP) and a time-dependent temperature model (∆T_*s*_) that describes changes in mean global surface temperature as a function of time or absolute temperature change potential (AGTP).

**Results:**

Straw-derived biodiesel reduced GWP by 33–80% compared with fossil fuels and primary fossil energy use for biodiesel production was 0.33–0.80 MJ_prim_/MJ, depending on the scenario studied. Simulations using the time-dependent temperature model showed that a scenario where all straw fractions were converted to energy carriers and no lignin was returned to soil resulted in the highest avoided climate impact. The SOC changes due to straw removal had a large impact on the results, both when using GWP and the time-dependent temperature model.

**Conclusions:**

In a climate perspective, it is preferable to combust straw lignin to produce electricity rather than returning it to the soil if the excess electricity replaces natural gas electricity, according to results from both GWP and time-dependent temperature modelling. Using different methods to assess climate impact did not change the ranking between the scenarios, but the time-dependent temperature model provided information about system behaviour over time that can be important for evaluation of biofuel systems, particularly in relation to climate target deadlines.

**Electronic supplementary material:**

The online version of this article (doi:10.1186/s13068-017-0907-9) contains supplementary material, which is available to authorised users.

## Background

The transport sector generates approximately 23% of energy-related global greenhouse gas (GHG) emissions [1], of which approximately 96% originate from fossil fuels [2]. Transportation fuels produced from biomass have been suggested as one measure to decrease the GHG emissions from transportation [1]. Biofuels are currently mainly produced from conventional feed and food crops. However, production of these feedstocks requires arable land and has therefore been criticised for competing with feed and food production [3] and for causing indirect land use changes that have been associated with large climate impacts [4, 5]. Biodiesel is an interesting fuel that is increasingly employed in Europe [6] and can be used in conventional cars in low blends with fossil diesel. At present, biodiesel is primarily produced from vegetable oils, but can be produced from several types of biomass including lignocellulose. Agricultural residues such as straw are promising alternative feedstocks for biofuel production [7], since they are associated with lower climate impact [8] and do not require extra land.

The environmental impact of ethanol from lignocellulosic biomass has been examined in numerous studies [8]. However, several process routes with multiple final products are possible, including biodiesel production from straw using oleaginous yeasts (lipid-accumulating yeasts). Organisms defined as oleaginous are capable of accumulating more than 20% of their dry weight as lipids and include bacteria, yeasts, filamentous fungi and algae [9]. Yeasts are promising organisms with their relatively fast growth rate, ability to grow in high cell densities, resistance to viral infection and the possibility to control bacterial contamination by using low pH conditions [10] and their ability to grow on multiple substrates [11]. Yeast upscaling to industrial scale is less complicated than for autotrophic microalgae, which is another organism considered for biodiesel production [12]. When the hemicellulose and cellulose in lignocellulosic biomass are hydrolysed, pentose and hexose sugars and weak acids such as acetic acid are formed. Many oleaginous yeasts can use pentoses and hexoses [13] and acetic acid [14] for accumulation of lipids, which is an advantage compared with the commonly used yeast for ethanol production (*Saccharomyces cerevisiae*) that requires metabolic engineering to convert pentoses or acetic acid to ethanol [15, 16].

Earlier studies on biofuels or bioenergy produced from lignocellulosic biomass clearly show that changes in carbon stocks, including living biomass and soil organic carbon (SOC), greatly affect the climate performance of biofuels [17–19]. For fuels produced from straw, changes in SOC due to straw removal have been proven to be important for climate performance [20–22]. Lignocellulosic biomass such as straw contains three main polymer fractions: cellulose, hemicellulose and lignin. The polysaccharides cellulose and hemicellulose can be converted to monosaccharides in a biochemical process and these sugars are used for subsequent production of fuels or other products. Lignin is a complex and irregular polymer that provides rigidity and resistance to decay in plants [23]. Although many applications for the lignin fraction have been considered [24], it usually represents a residue of biofuel production processes and is burned to provide heat and power for the biorefinery process.

Lignin is resistant to microbial degradation and only a few organisms can decompose it [23], but formation of stable SOC is complex and the contribution of lignin to SOC is currently under discussion [25]. Returning parts of the lignin to soil could be one strategy to decrease the impact on SOC and thereby improve the climate impact of biofuel production. Maintaining SOC levels has many significant functions for soil ecosystem services, and thereby agricultural productivity [26]. These impacts were not assessed in the present study, but are of significant importance for the long-term sustainability of agricultural systems.

Changes in SOC stocks are long-term processes that occur over several years [27]. In life cycle assessment (LCA) methodology, it is usually assumed that all emissions associated with the production of a product or service occurs as a pulse emission and no distinction is made on when in time the emissions occur. Changes over several years, such as SOC changes, are therefore often handled by allocating emissions [28] or estimating the average change over a selected period (see e.g. [17, 22, 29]). The time period considered has a large impact on the results [22]. For describing climate change impact, a widely used method in LCA is global warming potential (GWP), expressed in CO_2_-eq. The GWP of a specific greenhouse gas (GHG) is calculated as the cumulative radiative forcing (CRF) caused by emission of the gas, integrated over a specific time horizon, relative to the CRF of carbon dioxide (CO_2_) integrated over the same period [30]. The somewhat arbitrary selection of time horizon for integrating radiative forcing in GWP (often 100 years) has been questioned, since it has a great impact on the results, giving high importance to short-lived gases when short time horizons are used compared with longer time horizons [30]. For studies on bioenergy systems, the use of GWP has been questioned since it cannot capture fluxes of GHGs over time [31].

Alternative methods to handle temporary changes in carbon storage for bioenergy systems have been developed [32]. Ericsson et al. [33] developed a time-dependent LCA method to assess the climate impact of bioenergy systems. This method describes changes in mean global surface temperature as a function of time, referred to as ∆T_*s*_ or absolute global temperature change potential (AGTP) by the IPCC [34], thereby capturing fluxes of GHGs, including SOC changes. To date, the methodology has been used only on perennial bioenergy crops and forest residues (see for example [18, 33]). The present study attempted to apply the methodology to annual cropping systems.

The present study is, to our knowledge, the first assessment of the climate impact of biodiesel produced from straw using oleaginous yeast. In addition, we attempted to include changes in SOC when assessing the climate impact of the biofuel, using a newly developed method. To understand the effect of SOC changes on the climate impact of biofuels over time, it is essential to evaluate any biofuels intended for use in climate change mitigation.

### Goal and scope

#### Goal

The aim of this study was to assess the climate impacts and energy balances of biodiesel production from straw using oleaginous yeast, and subsequent biogas production from the residues. Particular emphasis was placed on how soil carbon changes over time affect the results. The study also explored whether parts of the lignin fraction of the biomass can be returned to the field to mitigate SOC changes.

#### Scope

This study builds on a previous study describing the process design and energy balance of biodiesel production from straw using oleaginous yeast [35]. That study included straw harvesting and transport, SOC changes due to straw harvesting and replacement of nitrogen removed with the straw, processing the straw in a biorefinery and production of biorefinery inputs. Building materials, other infrastructure and distribution of the products were not included. Since uptake of carbon and emission of carbon take place during the same year, CO_2_ emissions during combustion of the biofuel were considered carbon neutral, with the exception of CO_2_ emissions from combusting the methanol component in the biodiesel. In the present study, methanol was assumed to be of fossil origin and therefore these emissions represent net addition of CO_2_ to the atmosphere, in contrast to the biogenic carbon in the biodiesel originating from the straw. Calculations were performed using the system boundaries presented above, but also the calculation procedure in the Renewable Energy Directive (RED) [36]. The RED calculations excluded nutrient replacement and SOC changes.

Two functional units were used, 1 kg of straw and 1 MJ biodiesel. The functional unit 1 kg straw allows for comparison of resource use efficiency between the scenarios and of the potential benefit of all products produced from the biomass, while the functional unit 1 MJ biodiesel enables comparison between the scenarios, but also with other energy products.

The study was performed as an attributional LCA (ALCA), for which data were selected to represent the current or near-term situation in Swedish conditions.

## Methods

Climate impact was analysed using two methods, global warming potential (GWP_100_) and the time-dependent climate impact methodology described by Ericsson et al. [33]. The time-dependent model accounts for the timing of emission (or uptake) of the three major greenhouse gases (CO_2_, nitrous oxide (N_2_O) and methane (CH_4_)) and estimates climate impact as temperature response over time. Yearly emissions are estimated over 100 years. The time-dependent modelling shows the result of processing 1 kg straw or producing 1 MJ biodiesel yearly during 100 years. For straw, the uptake and release of CO_2_ in living biomass occurs during 1 year, and therefore these changes were not accounted for. However, changes in SOC due to straw harvesting occur over several years and were estimated by modelling.

When presenting the results per MJ biodiesel, the impact from the process was allocated to the different products based on lower heating value (LHV).

The GWP calculations included the same gases as the time-dependent modelling using characterisation factors in the IPCC report (2013) (Table 1) [37]. Soil organic carbon changes were included in these calculations using the average change in SOC over 100 years.

**Table 1:** Characterisation factors for GWP_100_

	GWP_100_ (kg CO_2_ eq/kg)
CO_2_	1
N_2_O	265
CH_4_	28

Three energy balance indicators were assessed: (1) Energy efficiency ratio (EE), calculated as ratio between the energy obtained and the energy in the feedstock (LHV), indicating the proportion of energy in feedstock converted to final product; (2) net energy ratio (NER), calculated as total primary fossil energy input/energy obtained (LHV), indicating the amount of fossil fuel used in production of the biofuel (values >1 indicate more fossil fuel is used than biofuels produced); and (3) fossil fuel replacement potential (FFRP), calculated by subtracting primary fossil fuel potentially replaced by the products from total use of primary energy in the whole production chain for 1 kg of dry matter (DM) straw input into the biorefinery. FFRP shows the potential fossil fuel replacement after taking the use of fossil fuels in the production chain into account.

### Reference system

The reference system consisted of conventional energy products equivalent to the energy products generated in the respective scenarios. When the FU 1 kg straw is used, different amounts of energy products are formed in the different scenarios. 1 MJ biodiesel was assumed to equal 1 MJ fossil diesel, 1 MJ biogas to equal 0.82 MJ fossil diesel [38] and 1 MJ electricity to equal 1 MJ electricity produced from natural gas.

Substitution effects were calculated by subtracting the impact of the reference system from the impact of the scenarios assessed. In the time-dependent modelling, the substitution effects describe the potential avoided warming effect if the products from the scenarios assessed were used instead of fossil fuels.

### Scenarios

**Fig. 1:**
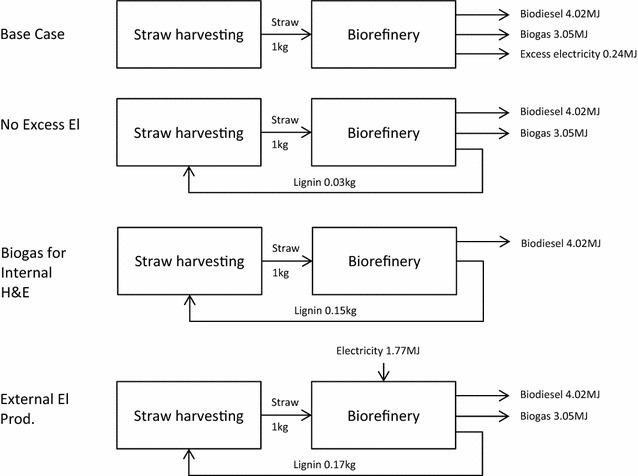
Illustration of the assessed scenarios. No lignin was returned to the field in the Base Case, where excess lignin was combusted to produce electricity (El). The flows presented in the figure are results also presented in Table 5. H & E = heating and electricity

The biorefinery process is explained in detail in Karlsson et al. [35]. In short, the straw was first pre-treated (steam explosion), followed by enzymatic hydrolysis and liquid–solid separation to separate out the solids to be combusted in the combined heat and power plant that supplies the plant with heat and electricity. The sugars from the enzymatic hydrolysis were fed into an aerobic reactor, where the lipid accumulation phase took place. The lipids were extracted without drying the yeast, using hexane. The extracted lipids were tranesterified to produce fatty acid methyl esters (FAME), here called biodiesel, while the yeast cell mass and other residues from the process were anaerobically digested to produce biogas. Four scenarios were included (Fig. 1). The ‘Base Case’ was based on the base case in Karlsson et al. [35] with a few changes (see Additional file 1). In that study, a number of process parameters were analysed, including lipid content, fermentation time and sugar concentration in the hydrolysate, and the results showed that increasing the lipid content was particularly beneficial for the energy balances assessed. However, the base case was considered the most likely scenario considering near-term technological development [35]. In the Base Case in the present study, all lignin and other residues were combusted to produce heat and electricity and the excess electricity was assumed to be sold. In order to save some of the lignin to return to the soil, three additional scenarios were assessed. In the ‘No Excess El’ scenario, only the lignin required to satisfy the electricity and heat demand of the process was combusted and the excess lignin was returned to the soil. In the ‘Biogas for Internal H&E’ scenario, the biogas and a minor fraction of the lignin were combusted to produce the heat and electricity required by the plant. Lastly, in the ‘External El Prod.’ scenario only the lignin required to produce the heat demand of the plant was combusted and the remaining lignin was returned to the soil. In this scenario, electricity required by the plant was assumed to be produced from natural gas (alternative electricity production forms were analysed in the sensitivity analysis).

### Life cycle inventory

Inputs throughout the whole process (Table 2), including harvesting and processing, were taken from Karlsson et al. [35]. Two changes were made to the input data. The enzymes used in this study were changed from Cellic ^®^ CTec3 to a new improved product, customised Cellic ^®^ 1.0 (both produced by Novozymes A/S). For the new enzyme product, the dose is higher (70%), while the environmental impact of the product is substantially lower (Table 2) (Jesper Kløverpris, personal communications April–July 2016). Furthermore, the nitrogen removed from the field with the straw was compensated for by adding mineral fertiliser assuming 0.5% N in straw [39], after accounting for the nitrogen content of the lignin-rich residue, which was assumed to be protein content divided by 6.25. The environmental impacts of the different inputs are presented in Table 3. ALCA data were used as much as possible, but data on enzymes were taken from a study that employed a consequential approach.

**Table 2:** Inputs used for straw harvesting and processing in the biorefinery, expressed per kg DM straw and per MJ produced

	Input per kg DM straw/per MJ produced				Unit
	Base case	No excess El	Biogas for internal H&E	External El Prod.	
Diesel (harvesting and transport)	0.34/0.05	0.34/0.05	0.34/0.08	0.34/0.05	MJ
Nitrogen compensation	5.00/0.68	4.88/0.69	4.33/1.08	4.27/0.60	g N
Sulphuric acid	2.40/0.33	2.40/0.34	2.40/0.60	2.40/0.34	g
Enzymes	20.49/2.80	20.49/2.90	20.49/5.09	20.49/2.90	g enzyme product
Ammonia	13.0/1.78	13.0/1.84	13.0/3.23	13.0/1.84	g
Hexane	6.46/0.88	6.46/0.91	6.46/1.61	6.46/0.91	g
Sodium hydroxide	1.35/0.18	1.35/0.19	1.35/0.33	1.35/0.19	g
Phosphoric acid	0.94/0.13	0.94/0.13	0.94/0.23	0.94/0.13	g
Methanol	12.5/1.71	12.5/1.77	12.51/3.11	12.5/1.77	g
External electricity	–	–	–	1.77/0.25	MJ

**Table 3:** Inputs used in the biorefinery and environmental impact data

Input	GWP (g CO_2_ eq)	Fossil energy (MJ)
Diesel (MJ)^a^	80.5	1.19
Nitrogen fertiliser (kg N)^b^	5630	48.9
Sulphuric acid (kg)^c^	123	2.12
Enzymes (kg product)^d^	985	12.8
Ammonia (kg N)^e^	2110	41.7
Hexane (kg)^f^	904	60.9
Methanol (kg)^g^	2210	37.4
NaOH^h^	2190	42.7
H_3_PO_4_^i^	1670	23.5
Electricity (natural gas) (MJ)^j^	121	1.88

^a^GWP calculated based on [40] and fossil energy use [41]

^b^Greenhouse gases and energy [42]

^c^[43] (process: sulphuric acid, liquid, at plant, RER)

^d^Personal communication, Jesper Kløverpris, 2016

^e^[43] (process: ammonia, liquid, at regional storehouse, RER)

^f^[43] (process: hexane, at plant, RER)

^g^ [43] (process: methanol, at plant, GLO) emissions of CO_2_ during combustion added

^h^ [43] (process: sodium hydroxide, 50% in H_2_O, production mix, at plant, RER)

^i^[43] (process: phosphoric acid, industrial grade, 85% in H2O, at plant, RER)

^j^Calculated based on [40] and 58% efficiency

### Soil carbon modelling

Soil carbon changes resulting from increased harvesting of straw were modelled over 100 years using the Introductory Carbon Balance Model (ICBM) [44]. A 5-year crop rotation was assumed [45] (Table 4). Straw was only harvested from the winter wheat, with 60% of the straw assumed to be harvested. Carbon inputs from straw, residues and roots were calculated using the function *C* = *a*+*sH*, where *a* and *s* are crop-specific parameters and *H* is the observed yield (as carbon) [46]. Yields were based on statistics giving the average winter wheat yield in 2015 for southern Sweden [47]. A yield increase of 800 kg/ha was assumed for wheat cultivated in the year after rapeseed [48]. Carbon content of the biomass was assumed to be 45% [49]. Lignin was assumed to be recycled to the field for all scenarios except the Base Case (Table 4). The lignin-rich residue constituted of approx. 73% lignin (dry matter basis), with the remainder being ash (13%), cellulose and hemicellulose (11%) and protein (2%). In the ICBM modelling only the lignin was accounted for, since the contribution of the other components to stable SOC is uncertain, especially after being subjected to steam explosion.

**Table 4:** Crop rotation and annual carbon (C) input from crop biomass residues and lignin residues

Crop sequence	Crop yield (*t* DM/ha)	Yearly C input to soil from biomass (*t* DM/ha)	Yearly C input from lignin residues (*t* DM/ha)
Winter wheat	6.4	3.4^a^	0/0.05/0.20/0.23^b^
Spring oilseed rape	1.8	2.9	
Winter wheat	8.3	3.6^a^	0/0.05/0.20/0.23^b^
Oats	4.7	3.4	
Winter barley	6.4	4.1	

^a^With 60% straw harvest

^b^Base Case/No Excess El/Biogas for Internal H&E/External El Prod

Initial soil carbon content (topsoil 0–25 cm) was estimated to 86 ton per hectare by running the model for 2000 years with the crop rotation presented in Table 4, with no straw harvest or lignin return.

ICBM is a two-compartment model considering one young (Y_*t*_) and one old (O_*t*_) soil carbon pool. The calculations were made using the following equations (from Eqs. 6 and 7 in [50]), adapted to handle three types of biomass inputs:

$$\text{Y}_{{[\text{AG, BG, LG}]\,t}} = (\text{Y}_{{[\text{AG, BG, L}]\,t - 1}} + \text{i}_{{[\text{AG, BG, LG}]\,t - 1}} )\,e^{{ - k_{y} r_{e} }}$$

$$\begin{aligned} O_{t} = \left( {O_{t - 1} - \left( {\frac{{h_{AG} *k_{y} }}{{k_{o} - k_{y} }}*\left( {Y_{AG t - 1} + i_{AG t - 1} } \right) + \frac{{h_{BG} *k_{y} }}{{k_{o} - k_{y} }}*\left( {\text{Y}_{BG t - 1} + i_{BG t - 1} } \right) + \frac{{h_{LG} .*k_{y} }}{{k_{o} - k_{y} }}*\left( {Y_{LG t - 1} + i_{LG t - 1} } \right)} \right)} \right) \hfill \\ *e^{{ - k_{o} r_{e} }} + \left( {\frac{{h_{AG} *k_{y} }}{{k_{o} - k_{y} }}*\left( {\text{Y}_{AG t - 1} + i_{AG t - 1} } \right) + \frac{{h_{BG} *k_{y} }}{{k_{o} - k_{y} }}*\left( {\text{Y}_{BG t - 1} + i_{BG t - 1} } \right) + \frac{{h_{LG} *k_{y} }}{{k_{o} - k_{y} }}*\left( {\text{Y}_{LG t - 1} + i_{LG t - 1} } \right)} \right)*e^{{ - k_{y} r_{e} }} \hfill \\ \end{aligned}$$

where *i*_AB_ is aboveground crop residues, *i*_BG_ is belowground crop residues, *i*_LG_ is lignin input (Table 4), *h*_AG_ (0.098), *h*_BG_ (0.23) and *h*_LG_ (0.52) are the humidification rate for aboveground residues, belowground residues and lignin, respectively, *r*_*e*_ (1.00 [44]) is a constant for climate (and edaphic) conditions and k_*y*_ (0.8 [44]) and k_*o*_ (0.00605 [44]) represent decomposition rate of the young and old pools. The humidification rates (*h*) for above- and belowground residues were estimated from the *h* value for the total plant biomass (0.125 [44]). Studies have shown that belowground crop residues contribute more to SOC than aboveground residues [51, 52], and it has been estimated that h for belowground residues is 2.3 higher than for aboveground residues [52]. Therefore, the mean value of h was calculated considering the above- and belowground biomass input to soil over the whole crop rotation without straw harvesting, assuming 2.3 times higher h for belowground residues. The value of *h*_LG_ was estimated from the h value for peat [52] and adjusted for more sandy soils using the h value for straw [44].

## Results

Production of energy carriers was highest in the Base Case (Table 5; Fig. 1). Biodiesel production was similar for all scenarios. Lignin was returned to the field in all scenarios except the Base Case.

**Table 5:** Production of energy carriers (biodiesel, biogas and excess electricity), use of externally produced electricity and return of lignin to the field for all scenarios

	Base case	No excess el	Biogas for internal H&E	External el prod.
Biodiesel (MJ/kg straw)	4.02	4.02	4.02	4.02
Biogas (MJ/kg straw)	3.05	3.05	–	3.05
Excess electricity (MJ/kg straw)	0.24	–	–	–
External electricity (MJ/kg straw)	–	–	–	1.77
Lignin returned to field (kg/kg straw)	–	0.03	0.15	0.17

### SOC modelling results

**Fig. 2:**
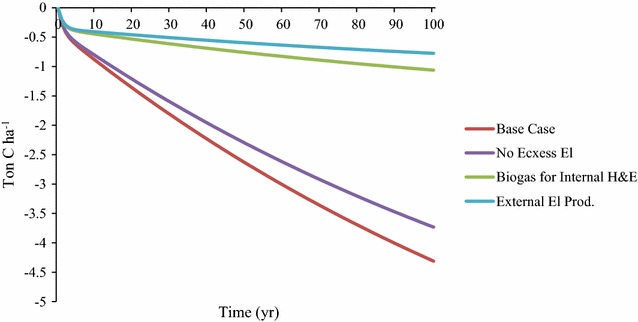
Soil organic carbon (SOC) losses (kg C per ha and year) in relation to the reference with no straw harvest

Soil carbon losses were highest in the beginning of the modelled time period, with 21, 22, 42 and 53% of the decrease in SOC occurring during the first 10 years for the Base Case, No Excess El, Biogas for Internal H&E and External El Prod scenarios, respectively (Fig. 2).

### Time-dependent climate model

**Fig. 3:**
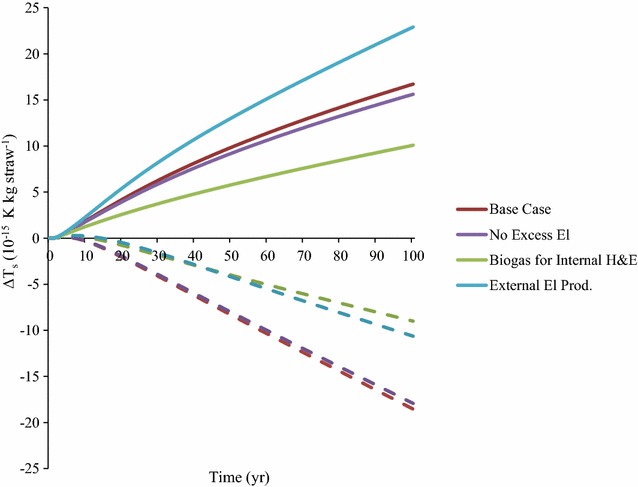
Time-dependent global mean surface temperature change (∆T_*s*_) from process emissions including harvesting, processing and soil organic carbon (SOC) changes due to straw harvesting for 1 kg straw (solid lines) and from replacement of equivalent products for each scenario (biodiesel and biogas replaced diesel and electricity replaced natural gas electricity) (dotted lines)

The Base Case showed a relatively high impact per kg straw (Fig. 3). However, in terms of potential avoided warming from replacing equivalent fossil products, the Base Case scenario showed the highest substitution potential, which was due to the higher energy output in this scenario. The difference between the Base Case and the No Excess El scenario was relatively small, but the results showed that it was beneficial to combust the excess lignin and produce electricity if this electricity replaces electricity produced from natural gas (Fig. 3).

The Biogas for Internal El scenario had the lowest impact per kg straw, but considering the possible substitution, this scenario has the lowest potential avoided warming due to the lower energy output (Fig. 3). Use of externally produced electricity resulted in high process impacts, as was seen for the External El Prod. scenario, despite the fact that this scenario returned most lignin back to the field. The impact was due to electricity produced from natural gas. For the External El Prod. scenario this resulted in relatively low avoided warming potential, despite the relatively high production of energy carriers (Table 5).

Potential avoided warming by replacing fossil alternatives was achieved after 7 years of operation for the Base Case and the No Excess El scenarios, after 12 years for the Biogas for Internal H&E scenario and after 15 years for the External El Prod. scenario (Fig. 3).

**Fig. 4:**
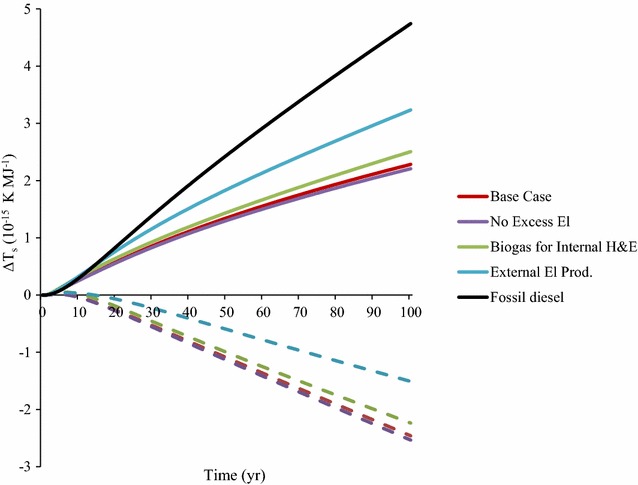
Time-dependent global mean surface temperature change (∆T_*s*_) showing allocated impacts for the process, including harvesting, processing and soil organic carbon (SOC) changes due to straw harvesting for 1 MJ biodiesel (solid lines) and potential avoided warming through substitution for fossil diesel (dotted lines)

The allocated results for 1 MJ biodiesel showed that the No Excess El scenario had the lowest impact per MJ produced and the highest potential avoided warming through substituting for fossil diesel (Fig. 4). The results were different from when the functional unit 1 kg straw was used. When using the functional unit 1 MJ biodiesel, the substitution effect only describes the effect of replacing equivalent amounts (1 MJ) of fossil diesel, whereas when using the functional unit 1 kg straw the substitution effect describes the effect of replacing multiple products (Fig. 3). When using the functional unit 1 MJ biodiesel in the Base Case (Fig. 4), the results do not show the benefit of replacing natural gas electricity, which is shown when the functional unit 1 kg straw is used (Fig. 3).

### GWP and energy balances

Compared with fossil fuels, based on MJ biodiesel produced the GWP was reduced from −33 to −80% depending on scenario and system boundaries (Table 6). The No Excess El scenario had the lowest GWP when SOC changes were included, because part of the lignin fraction was returned to the soil in this scenario, which lowered the SOC loss compared with the Base Case. For all scenarios, SOC changes and nutrient replacement due to straw harvesting greatly influenced the results and when these effects were included, the GWP reduction potential compared with fossil fuels was −33 to −54%, whereas it was 41–80% when these effects were not included (GWP RED) (Table 6). The SOC changes had the largest impact on the Base Case and No Excess El scenarios, since no lignin was returned in the Base Case and a minor fraction of lignin was returned in the No Excess El scenario, which resulted in the highest SOC losses for these scenarios. Calculating GWP using the RED methodology resulted in 41–80% reductions compared with fossil fuels (Table 6). All scenarios except External El Prod. complied with the forthcoming 60% reduction target in RED [36].

**Table 6:** Effects of the different scenarios on climate change calculated using global warming potential (GWP) and the Renewable Energy Directive (GWP RED) approach and value of the net energy ratio (NER), energy efficiency (EE) and fossil fuel replacement potential (FFRP) energy balance indicators

	Base case	No excess el	Biogas for internal H&P	External el prod.	Fossil diesel
GWP (g CO_2_eq/MJ)^b^	38.5 (−52%)	37.2 (−54%)	42.4 (−47%)	53.9 (−33%)	
GWP RED (g CO_2_eq/MJ)^b^	16.3 (−80%)	16.9 (−79%)	28.1 (−65%)	47.1 (−41%)	
NER (MJ_prim_/MJ)	0.33	0.34	0.59	0.80	1.19
EE (%)	41%	40%	22%	40%	
FFRP (MJ/kg straw)	−5.81	−5.36	−2.42	−2.07	

^a^Values in brackets are reduction potential relative to fossil fuels

The Base Case had the lowest primary fossil input per MJ biodiesel (NER) and the highest energy conversion efficiency (EE) due to its higher energy output. FFRP was also highest for the Base Case.

**Fig. 5:**
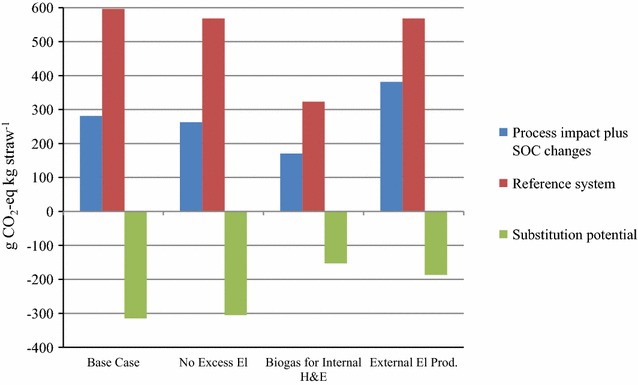
Global warming potential (GWP) per kg straw for the biorefinery process, including soil organic carbon (SOC) changes, the reference system and the substitution potential when the biodiesel, biogas and electricity replace equivalent fossil products

Figure 5 shows the climate impact in GWP with the functional unit 1 kg straw and the climate impact of producing an equivalent amount of fossil energy (reference system). Since the amount of energy carriers produced in the different scenarios differed, the reference systems also differed between the scenarios. The substitution potential was calculated by subtracting the impact of the reference system from the process impacts (including SOC changes). The largest GWP substitution potential was found for the Base Case. Regarding the ranking of the different scenarios, the results were the same as those for the time-dependent climate modelling (see Fig. 3).

**Fig. 6:**
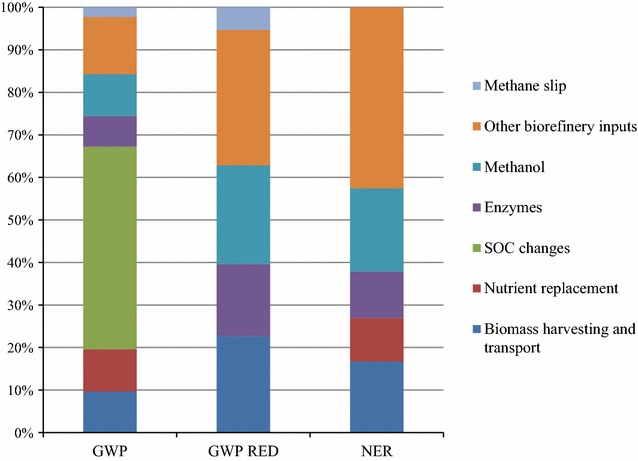
Relative contribution of different life cycle steps to the total climate impact (GWP and GWP RED) and primary energy use (NER) for the Base Case

The relative contribution to the climate impact and primary energy use from different life cycle steps for the Base Case is shown in Fig. 6. As can be seen, SOC changes represented the single most important impact, contributing 48% of the total GWP. Nutrient replacement contributed 10% to GWP and the use of enzymes for enzymatic hydrolysis 7–17%. Other biorefinery inputs were also important contributors, with ammonia for yeast propagation being the main contributor in this category.

## Sensitivity analysis

Five sensitivity analyses were performed, where the following was changed:

1. The variable h_LG_ in SOC modelling (±20%).
2. Input data on mineral fertiliser production to represent best available technology (BAT) with N_2_O cleaning (data from [42]).
3. External electricity production in the External El Prod. scenario to (a) lignite and (b) straw (data from [42]).
4. Conversion efficiency for biogas production in the Base Case (±10%).
5. The time horizon over which SOC changes are allocated (only applicable for the GWP calculations), from 100 years to: (a) 50 years, (b) 25 years and (c) 10 years.

Results from the sensitivity analysis for the time-dependent modelling are presented in Table 7. The results for GWP were largely similar to those for the time-dependent modelling, and are presented in Additional file 1: Table S2. Changing *h*_LG_ gave a larger impact for the scenarios returning higher amounts of lignin residues (the Biogas for Internal H&E and External El Prod. scenarios). Mineral fertiliser production was initially estimated using the average impact from European nitrogen fertiliser production (Table 7), but in the sensitivity analysis this was changed to BAT (data from [42]) which increased the avoided warming potential (Table 7). The results for the External El Prod. scenario were greatly affected by changing the source of external electricity production, both when using the time-dependent (Table 7) and GWP (Additional file 1: Table S2) method. The time horizon over which the SOC changes were allocated (sensitivity analysis 5a–c presented in Additional file 1: Table S2) also greatly influenced the results. The use of 25 years, which is similar to the 20 years used in the ILCD handbook [28], increased the GWP by 23–10%, while a 10-year time horizon increased GWP by 63–27%.

**Table 7:** Sensitivity analysis showing the relative changes from the initial analysis of final temperature response ΔT_*s*_ at year 100 in 10^−15^ K for annual processing of 1 kg straw

	Base case	No excess el	Biogas for internal H&E	External el prod.
Initial analysis	−18.6	−17.9	−9.0	−10.6
h_LG_ value	NA	±1%	±10%	±12%
Mineral fertiliser impact	+4%	+4%	+6%	+5%
Electricity from lignite	NA	NA	NA	−161%
Electricity from straw	NA	NA	NA	116%
Biogas production	±7%	NA	NA	NA

A positive change indicates greater avoided warming potential (*NA* not applicable)

## Discussion

### Comparison of GHG performance to other bio-based diesel production

In the present study, all scenarios had a lower climate impact than fossil fuels when using both 1 MJ and 1 kg straw as the functional unit (Table 6; Fig. 5). The GWP of biodiesel produced from straw using oleaginous yeast was found to be 16–54 g CO_2_/MJ biodiesel, depending on scenario and system boundaries.

Several other studies have been performed on diesel fuels produced from other biomass and using different conversion technologies [53]. Rapeseed methyl ester (RME) is the most common feedstock in Europe [54] and has been studied in numerous LCA studies, with a review reporting results varying from 40 ± 2 g CO_2_ eq/MJ RME [53]. For Swedish conditions, climate impact has been estimated to be 36–46 g CO_2_ eq/MJ [55], while the default value in RED is set to 52 g CO_2_ eq/MJ [36].

A challenge when comparing LCA studies is how land use is handled in the different assessments. In the present study, the impact from harvesting of straw was included (SOC changes and nitrogen replacement). The equivalent change for dedicated energy crops such as rapeseed would be to include direct land use changes when cultivating rapeseed, which could either decrease or increase the carbon stock depending on previous land use [56]. Malça et al. [56] found that when land use change from two reference land uses (one with low carbon input and full tillage and one with high carbon input and reduced tillage) was accounted for, the results varied from 48 to 185 g CO_2_ eq/MJ biodiesel [56]. Dedicated energy crops could also be associated with indirect land use change, which could increase the climate impact significantly [5]. This was not included in the above-mentioned studies.

Other challenges when comparing studies are differences in the technical production system and in the background system with different cultivation systems, electricity mixes etc. Furthermore, the method used to allocate emissions between RME and the main co-product rapeseed cake (which can be used as a protein feed) has been proven to be influential for the results [56, 57]. For the assessment of straw biodiesel, the method for allocating emissions between straw and grain is important [58].

Although there have been techno-economic analyses of biodiesel production from lignocellulose using oleaginous yeast [59, 60], we could not find any previous calculations on the climate impact of this fuel. Biodiesel can also be produced from lignocellulosic biomass using gasification and subsequent catalytic conversion of the syngas to diesel-like fuels, e.g. Fisher-Tropsch (FT) diesel. In a review, GWP for biodiesel production from gasification of straw and miscanthus was found to be 37 ± 11 g CO_2_ eq/MJ fuel [53]. The GWP for biodiesel production from wood has been estimated to be 19 ± 6 g CO_2_ eq/MJ fuel [53]. As for agricultural soils, the use of residues from forestry, including tops, branches and stumps, is also associated with carbon storage changes that will affect the GHG balance of the fuel [61, 62].

A thorough discussion on the energy balance of bio-based diesel fuels can be found in Karlsson et al. [36].

### Alternative uses of lignocellulosic biomass from forest residues and straw

Gustavsson et al. [63] assessed the climate effects over time of using forest residues for a number of different energy purposes, including heat and electricity and biodiesel. They found that replacing coal was far more beneficial than replacing fossil diesel, due to the relatively inefficient biomass to biofuel conversion pathway for biodiesel compared with biomass to heat and electricity production. They argue that as long as coal and other fossil fuels are used for electricity production, biomass would be more efficiently used there [63]. In Sweden, in contrast to many other countries, electricity production has a relatively low share of fossil fuels. Therefore in a Swedish perspective lignocellulosic biomass might be more efficiently used in the transport sector than in the electricity and heating sector. However, when discussing climate impact mitigation, a European or global perspective is far more relevant than a strictly Swedish perspective. In addition, it is important to consider the options for the different energy demanding sectors. New renewable electricity production methods are continually being developed and increasingly used, with wind and solar energy expanding most rapidly [64]. In the transport sector, however, in the short to mid-term perspective growth in liquid biofuels is expected to remain stable, mainly due to low blend mandates [64]. Low environmental impact biofuels need to be developed for this purpose, preferably from feedstock that does not require extensive use of arable land.

In the present study, the lignin in straw was used to produce electricity or as a soil amendment. Several alternative uses of lignin have been proposed, including for example in dyes, synthetic floorings, paints and fuels [65]. Some of these applications, such as synthetic flooring, would temporarily sequester the carbon from the lignin (with the retention time depending on lifetime of the material and waste management practices). This differs from using lignin as a soil amendment, when part of the carbon is retained in the soil for a longer time, while a large fraction is released into the atmosphere as CO_2_ during the first  years. Using lignin for an application that temporarily sequesters the carbon would clearly affect the time-dependent climate impact and (temporarily) lower the GWP. However, SOC losses would be similar to those in the Base Case (Fig. 2) if no lignin residues were returned to the soil.

### Method for including carbon stock changes in LCA

By using the time-dependent temperature model, the choice of two rather arbitrary time horizons is partly avoided, i.e. the time horizon for the CRF used to estimate GWP for different gases (commonly 100 years is used) and the period over which SOC change is considered. For SOC changes, in this study, the GWP increased by 3–11% when the time changed from 100 to 25 years and by 27–63% when the time changed to 10 years, illustrating that this choice has potentially a great impact on the results. When using a single score climate indicator and when emissions vary from year to year, such as SOC changes, several questions arise. In the SOC modelling, it was found that SOC losses were highest in the beginning (21–53% of the losses occurred during the first 10 years) and then decreased (Fig. 6). The question in this case is whether the straw harvested in the first years should carry most of the burden from SOC change or, since the lower SOC change after 10 years of straw harvest is due to the higher SOC losses earlier, whether the burden should be more evenly distributed over a longer time. There is no commonly agreed method to handle this in LCA, which is problematic since these effects can have a great influence on the climate impact.

Compared with a single score indicator such as GWP, the time-dependent model can give additional information such as the shape of the climate impact and the behaviour of the system over time [32]. This study focused on annual crops and therefore changes in C stock in living biomass were not included, resulting in lower annual volatility than for perennial crops [18, 33]. However, the SOC changes are not the same every year and the time-dependent modelling gives new insights into how SOC changes affect climate impact over time. For example, in this study the modelling showed when in time the systems studied could result in potential avoided warming if fossil fuels were replaced.

Using time-dependent modelling or GWP did not change the ranking between the different scenarios either when using 1 kg straw or 1 MJ biodiesel as the functional unit. Therefore, the conclusions on the scenarios that were least or most preferable in a climate perspective would not change when using these different methods. However, the additional information about system behaviour over time could change the evaluation of the biofuel systems, in particular in relation to climate target deadlines.

The GWP concept, with all its limitations, also has some advantages [30]. It is a well-established method that is used in most LCA studies and therefore using this indicator enables comparison of results of different studies. The GWP metric is also politically feasible and has been broadly debated, so that strengths and weaknesses are rather well-known, although perhaps not equally well by all users of GWP, such as policymakers [30]. Considering the additional information that can be achieved using the time-dependent method, it could serve as an important complement to characterisation factor-based climate impact indicator [33] such as GWP.

It is important to note when including SOC changes in calculation of the climate impact of biofuels and other bio-based products that the modelling of SOC changes is associated with large uncertainties. In the present study, the humidification rate of the lignin residues from the process, which basically describes how much the lignin fraction contributes to the stable SOC, was not known because to our knowledge, there have been no long-term experiments on application to soil of relatively pure lignin. The effect of changing the h value for lignin by only ±20% was assessed in a sensitivity analysis and was found to affect the final results for the whole production system by ±1 to 12% for the time-dependent model and GWP results, with the largest impact for the Biogas for Internal H&P scenario due to the relatively high lignin return and relatively low total impact. Furthermore, it should be noted that SOC changes have effects on agricultural systems that were not included here and are not included in most LCA studies. These effects include changes in water-holding capacity, erosion etc., all of which can have effects on the future sustainability of food production [26], e.g. in maintaining high yields.

## Conclusions

The climate impact of biodiesel produced from oleaginous yeast was found to be 16.3–53.9 g CO_2_/MJ and primary fossil energy use 0.33–0.80 MJ_prim_/MJ biodiesel, depending on scenario and system boundaries. In a climate perspective, it was found using both GWP and the time-dependent modelling that it was preferable to combust the lignin in a combined heat and power plant instead of returning it to the soil if the excess electricity replaced natural gas electricity.

Comparing the climate impact found with that in other studies was problematic because of the many different methods used to handle land use and land use change in LCA. However, compared with other published results, the straw-based biodiesel produced in this study (including SOC changes) was largely similar to biodiesel produced from rapeseed, although including direct or indirect land use effects in rapeseed biodiesel studies would greatly affect the results.

When using the functional unit 1 MJ biodiesel, avoided warming potential was higher for the No Excess El than the Base Case scenario, because the potential avoided warming from replacing natural gas electricity was not included when this functional unit was used. Furthermore, the GWP was higher for the Base Case than for the No Excess El scenario when SOC changes are included. GWP was lowest for the Base Case when calculated using the RED methodology.

Using time-dependent modelling or GWP resulted in the same ranking of the different scenarios. However, the additional information obtained when using the time-dependent model about system behaviour over time could change the evaluation of the biofuel systems, in particular in relation to climate target deadlines.

## Additional file

**Additional file 1.** Additional information for the article.

## Abbreviations

∆T_*s*_: mean surface temperature change
AGTP: absolute temperature change potential
ALCA: attributional LCA
BAT: best available technology
CRF: cumulative radiative forcing
DM: dry matter
EE: energy yield
FAME: fatty acid methyl esters
FFRP: fossil fuel replacement potential
FT diesel: Fisher-Tropsch diesel
GHG: greenhouse gas
GWP: global warming potential
ICBM: introductory carbon balance model
LCA: life cycle assessment
LHV: lower heating value
NA: not applicable
NER: net energy ratio
RED: renewable energy directive
RME: rapeseed methyl esters
SOC: soil organic carbon
