# Supplementary material for: Greenhouse gas performance of biochemical biodiesel production from straw: soil organic carbon changes and time-dependent climate impact
Source: Biotechnol Biofuels. 2017 Sep 13;10:217. doi: 10.1186/s13068-017-0907-9 (PMC5598076; doi:10.1186/s13068-017-0907-9)
Supplement: Supplementary file 1 — Additional file 1. Additional information for the article. [file 13068_2017_907_MOESM1_ESM.docx]

**Supporting information for the article: Greenhouse gas performance of biochemical biodiesel production from straw - soil organic carbon changes and time-dependent climate impact**

Changes from Karlsson et al. [1].

- Nitrogen compensation for the nitrogen removed with the straw was included.
- Changes to combined heat and power (CHP) plant. The steam was mixed with lower temperature water from superheated to saturated steam before being used in the process.
- The fraction of protein and extractives remaining in the solid fraction after L/S separation after hydrolysis was assumed to be 20% and 66%, respectively.
- Life cycle inventory data for enzymes were changed (see main manuscript Table 2).

Table S1. Energy demand for unit processes with gross heating and cooling requirements (req.) for the Base Case

| Unit process | Electricity req. | Heat req. | Cooling req. | Comment | Reference |
| --- | --- | --- | --- | --- | --- |
| ***Pre-treatment*** |  |  |  |  |  |
| Size reduction | 0.0096 kWh/kg biomass (DM)  Total: 60 kW/h |  |  |  | [2] |
| Straw feeding system | 0.0071 kWh/kg straw  Total: 52 kW/h |  |  |  | [3] |
| Pre-treatment reactor |  | 0.36 kWh/kg DM straw  Total: 2220 kW/h |  |  | Own calculations |
| Flash | 1.643E-4 kWh/kg outflow  Total: 2.13 kW/h |  |  |  | [3] |
| Heat exchange after pre-treatment |  |  | Total: 1666 kW/h |  | Process model |
|  |  |  |  |  |  |
| ***Hydrolysis*** |  |  |  |  |  |
| Pump | Total: 0.59 kW/h |  |  |  | Process model |
| Cooling of hydrolysate |  |  | Total: 350kW/h |  |  |
| Hydrolysis reactor | 0.03 kWh/m3 active volume  Total: 41.7kW/h | Total: 378 kW/h |  |  | Power: [3]  Heat: Process model |
| L/S separation (whole Pneumpress incl. SEP block) | 2.0E-3 kWh/kg solids  Total: 4.78 kW/h |  |  | 2 % losses of soluble sugars to the solid fraction | Power: [3]  Losses: own calculations |
| Pump to L/S separation | Total: 4.44 kW/h |  |  |  | Process model |
| Air compressor for flash dryer | Total: 47.51 kW/h |  |  |  | Process model |
| Cooling air |  |  | Total: 44 kW/h |  | Process model |
|  |  |  |  |  |  |
| ***Lipid accumulation and yeast growth*** |  |  |  |  |  |
| Pump hydrolysate | Total: 1.24 kW/h |  |  |  | Process model |
| Cooling of hydrolysate |  |  | Total: 324 kW/h |  |  |
| Aeration and agitation of fermenters | 0.611 kW/m3 active volume  Total:  Lipid acc: 1742 kW/h  Yeast growth: 259 kW/h |  | Total:  Lipid acc: 3776 kW/h  Yeast growth: 2708 kW/h | Cooling: based on energy content of reactants and energy demand for agitation | Power: [4]  Cooling: own calculations |
| Cooling air |  |  | Total: 90 kW/h |  |  |
| Pump yeast | Total: 0.59 kW/h |  |  |  | Process model |
|  |  |  |  |  |  |
| ***Lipid extraction*** |  |  |  |  |  |
| Pressure filter | 0.88 kWh/m3  Total: 21.2 kW/h |  |  |  | [5] |
| Heating for deactivation of enzymes |  | Total: 194 kW/h |  |  | Process model |
| Homogeniser | 37 kWh/m3  Total: 186 kW/h |  |  |  | [6] |
| Cooling of yeast mass |  |  | Total: 75 kW/h |  | Process model |
| Mixer | 3.3 kW/m3 mixed volume, residence time 50min  Total: 13.8 kW/h |  |  |  | [6] |
| Purification | 30 kWh/ton unpurified oil  Total: 20.2 kW/h | 137 kWh/ton unpurified oil  Total: 92 kW/h |  |  | [7] assuming 90% efficiency of heat boiler |
| Evaporator |  | Total: 860 kW/h |  |  | Process model |
| Cooling recycled hexane |  |  | Total: 895 kW/h |  | Process model |
|  |  |  |  |  |  |
| ***Transesterification*** |  |  |  |  | Process model |
| Pump for lipids | Total: 0.28 kW/h |  |  |  | Process model |
| Pump for methanol | Total: 0.07 kW/h |  |  |  |  |
| Reactor |  |  |  | 99% conversion |  |
| Methanol col. |  | Total: 90 kW/h | Total: 92 kW/h |  | Process model |
| Pump to wash column | Total: 0.09 kW/h |  |  |  | Process model |
| Wash column |  |  |  |  |  |
| Ester column |  | Total: 248 kW/h | Total: 192 kWh/h |  | Process model |
| Glycerol column |  | Total: 61 kW/h | Total: 52 kW/h |  | Process model |
|  |  |  |  |  |  |
| ***WWT including biogas*** |  |  |  | Largely modelled as Area 600 in [3] except for biogas upgrading |  |
| Heater prior to AD |  | Total: 182 kW/h |  |  | Process model |
| Pump2 | Total: 1.97 kW/h |  |  |  | Process model |
| AD reactor | 0.009 kWh/m3  Total: 35 kW/h | Total: 53 kW/h |  |  | Electricity:[3]  Heat: Process model |
| Pump to filterpress 1 | Total: 6.3 kW/h |  |  |  | Process model |
| Filterpress 1 | 1.12E-4 kWh/kg inflow  Total: 2.96 kW/h |  |  |  | [3] |
| Aerobic reactor | 0.041 kWh/kg O2 consumed during degradation  Total: 244 kW/h |  | Total: 617 |  | Electricity:[3]  Cooling: Process model |
| Pump | Total: 1.25 kW/h |  |  |  | Process model |
| Clarifier | 2.14E-5 kWh/kg inflow  Total: 0.52 kW/h |  |  |  | Process model |
| Polymer addition | 0,0014 kWh/kg inflow  Total: 1.40 kW/h |  |  |  | [3] |
| Pump to filterpress 2 | 0.45 kW/h |  |  |  | Process model |
| Filterpress 2 | 1.12E-4 kWh/kg inflow  Total: 0.12kW/h |  |  |  | [3] |
| Mechanical cleaning | 4.29 E-5 kWh/kg inflow  Total: 0.011 kW/h |  |  |  | [3] |
| Biogas upgrading | 0.60 kWh/m3 upgraded biogas  Total: 292 kW/h |  | Total: 216 kW/h |  | Own estimations based on [8] |
|  |  |  |  |  |  |
| ***Combustion*** |  |  |  | Modelled as Area 800 in [3] see also [9] | [3, 9] |
| Heat |  | Generated: 4216 kW/h |  |  |  |
| Power | Used: 129 kW/h  Generated: 3583 kW/h  Total use in the plant: 3173 kW/h  Excess electricity: 410 kW/h |  |  |  |  |

**Sensitivity analysis**

The sensitivity analysis are further described in the main paper, they were as follows:

1. The variable h_LG_ in the SOC modelling (±10%)
2. Input data on mineral fertiliser production to represent best available technology (BAT) with N_2_O cleaning.
3. External electricity production in the External El Prod. scenario to a) lignite and b) straw.
4. Conversion efficiency for biogas production in the Base Case (±10%).
5. The time horizon over which SOC changes are allocated (only applicable for the GWP calculations), from 100 years to: a) 50 years, b) 25 years and c) 10 years.

Table S2. Sensitivity analysis showing relative change from the initial analysis (in g CO_2_ eq MJ^-1^), where a positive change indicates an increase in GWP (NA= not applicable)

|  | Base Case | No Excess El | Biogas for Internal H&E | External El Prod. |
| --- | --- | --- | --- | --- |
| Initial analysis | 38.51 | 37.2 | 42.4 | 53.9 |
| 1. | NA | ±1% | ±12% | ±6% |
| 2. | -4% | -4% | -6% | -2% |
| 3a. | NA | NA | NA | +78% |
| 3b. | NA | NA | NA | -53% |
| 4. | ±4% | NA | NA | NA |
| 5 a. | +11% | +10% | +8% | +3% |
| 5 b. | +23% | +23% | +23% | +10% |
| 5 c. | +51% | +53% | +63% | +27% |

**References:**

1. Karlsson H, Ahlgren S, Sandgren M, Passoth V, Wallberg O, Hansson P-A. A systems analysis of biodiesel production from wheat straw using oleaginous yeast: process design, mass and energy balances. Biotechnology for Biofuels. 2016,9(1): **DOI:** 10.1186/s13068-016-0640-9.

2. Miao Z, Grift TE, Hansen AC, Ting KC. Energy requirement for comminution of biomass in relation to particle physical properties*.* Industrial Crops and Products. 2011;33(2):504-513.

3. NREL. Bioethanol from corn stover process. A sample modell provided by Aspen Tech. National Renewable Energy Laboratory. 2006.

4. Hensirisak P, Parasukulsatid P, Agblevor FA, Cundiff JS, Velander WH. Scale-up of microbubble dispersion generator for aerobic fermentation*.* Applied Biochemistry and Biotechnology. 2002;101(3):211-227.

5. Grima EM,Helarbi E-H, Ferández AFG, Medina RA, Chisti Y. Recovery of microalgal biomass and metabolites: process options and economics. Biotechnology advances. 2003;20(7):491-515.

6. Stephenson AL, Kazamia E, Dennis JS, Howe CJ, Scott SA, Smith AG. Life-cycle assessment of potential algal biodiesel production in the United Kingdom: a comparison of raceways and air-lift tubular bioreactors. Energy & Fuels. 2010;24(7):4062-4077.

7. Stephenson A, Dennis J, Scott S. Improving the sustainability of the production of biodiesel from oilseed rape in the UK. Process Safety and Environmental Protection. 2008;86(6):427-440.

8. Cozma P, Ghinea C, Mămăligă I, Wukovits W, Friedl A, Gavrilescu M. Environmental impact assessment of high pressure water scrubbing biogas upgrading technology. CLEAN–Soil, Air, Water. 2013;41(9):917-927.

9. Humbrid D, Davis R, Tao L, Kinchin C, Hsu D, Aden A, Schoen P, Lukas J, Olthof B, Worley M, Sexton D, Dudgeon D. Process Design and Economics for Biochemical Conversion of Lignocelluloic Biomass to Ethanol Dilute-Acid Pretreatment and enzymatic Hydrolysis of Corn Stover. Technical Report NREL/TP-5100-47764. National Renewable Energy Laboratory. 2011.
